# Magnetic Resonance Spectroscopy Facilitates the Understanding of the Pathophysiology of Cerebellar Arteriovenous Malformations

**DOI:** 10.7759/cureus.68052

**Published:** 2024-08-28

**Authors:** Hiroaki Kubo, Hattori Kenichi, Hisashi Hatano, Shigeru Fujitani

**Affiliations:** 1 Neurosurgery, Japanese Red Cross Aichi Medical Center Nagoya Daiichi Hospital, Nagoya, JPN

**Keywords:** pathophysiology, n-acetylaspartate, magnetic resonance spectroscopy, venous congestion, arteriovenous short-circuit disease

## Abstract

Magnetic resonance spectroscopy (MRS) is a non-invasive imaging technique that facilitates the observation of tissue metabolism. It holds potential not only in research but also in clinical settings for understanding pathophysiology. This report presents the case of a 73-year-old woman with cerebellar arteriovenous malformation (AVM) in which MRS contributed to understanding the condition. Preoperative magnetic resonance imaging revealed T2/fluid-attenuated inversion recovery hyperintensity in the right cerebellar hemisphere. MRS of the same site showed an increase in lactate (Lac) and a decrease in N-acetylaspartate (NAA) levels. Through examination, she was diagnosed with a micro-AVM. Although transarterial embolization was performed on another day, completely occluding the shunt and treating the AVM, MRS showed a persistent decrease in NAA and elevated Lac levels and suggested that irreversible brain tissue damage had occurred due to the progression of venous congestion. The use of MRS in patients with suspected cerebellar AVMs allows for evaluating the degree of brain damage due to venous congestion, providing valuable insights for treatment decisions, in addition to evaluating treatment outcomes.

## Introduction

Magnetic resonance spectroscopy (MRS) is an imaging technique that allows for the quantification of brain tissue metabolites without the administration of tracers [1] used primarily in clinical settings for the differential diagnosis of brain tumors. There is a report suggesting that MRS may be useful for preoperative evaluation of whether edema associated with symptomatic meningiomas is reversible after tumor resection [2]. Among the various metabolites detected by MRS, the most significant changes observed in cerebrovascular disorders are an increase in lactate (Lac), resulting from anaerobic glycolysis due to a reduced oxygen supply, and a decrease in N-acetylaspartate (NAA), resulting from impaired energy metabolism within the mitochondria [3]. Brain arteriovenous shunt disease is classified by Borden et al. [4] and Cognard et al. [5]. Cortical venous reflex (CVR) is considered a predictor of intracranial hemorrhage or non-hemorrhagic neurological deficits. If hemorrhage occurs, surgical treatment may be considered, but for cases with only CVR, there are no clear indicators for treatment decisions. Although there are reports of the use of MRS in the field of cerebral vascular disorders, particularly in acute cerebral ischemia, there is limited documentation on its application in venous circulatory disturbances.

In this article, we report a case of cerebellar arteriovenous malformation (AVM) where MRS was useful for understanding the pathology. Pre-treatment MRS showed a decrease in NAA and an increase in Lac. After treatment, although the patient’s dysarthria and ataxia improved, MRS continued to show a decrease in NAA and high levels of Lac, suggesting that brain tissue necrosis had occurred due to venous congestion.

## Case presentation

Case 1

Presentation

The patient was a 73-year-old woman with chief complaints of dysarthria and dizziness. Her medical history included a dissecting thoracic aortic aneurysm and hypertension. There was no relevant family history, with no history of conditions such as hereditary hemorrhagic telangiectasia.

The patient had a one-month history of progressive worsening of dysarthria and dizziness and sought medical attention locally. On magnetic resonance imaging (MRI), T2/fluid-attenuated inversion recovery (FLAIR) hyperintensity and a mass effect were seen in the right cerebellar hemisphere, suggesting a brain tumor. She was then referred to the neurosurgery department for further evaluation.

Neurological Examination

On the initial neurological examination, no double vision/nystagmus, normal eye movements, no facial nerve paralysis, midline tongue protrusion, impaired lingual movements, no limb paralysis, slight instability in upright posture, and a slightly widened gait during walking were noted.

Imaging

**Figure 1:**
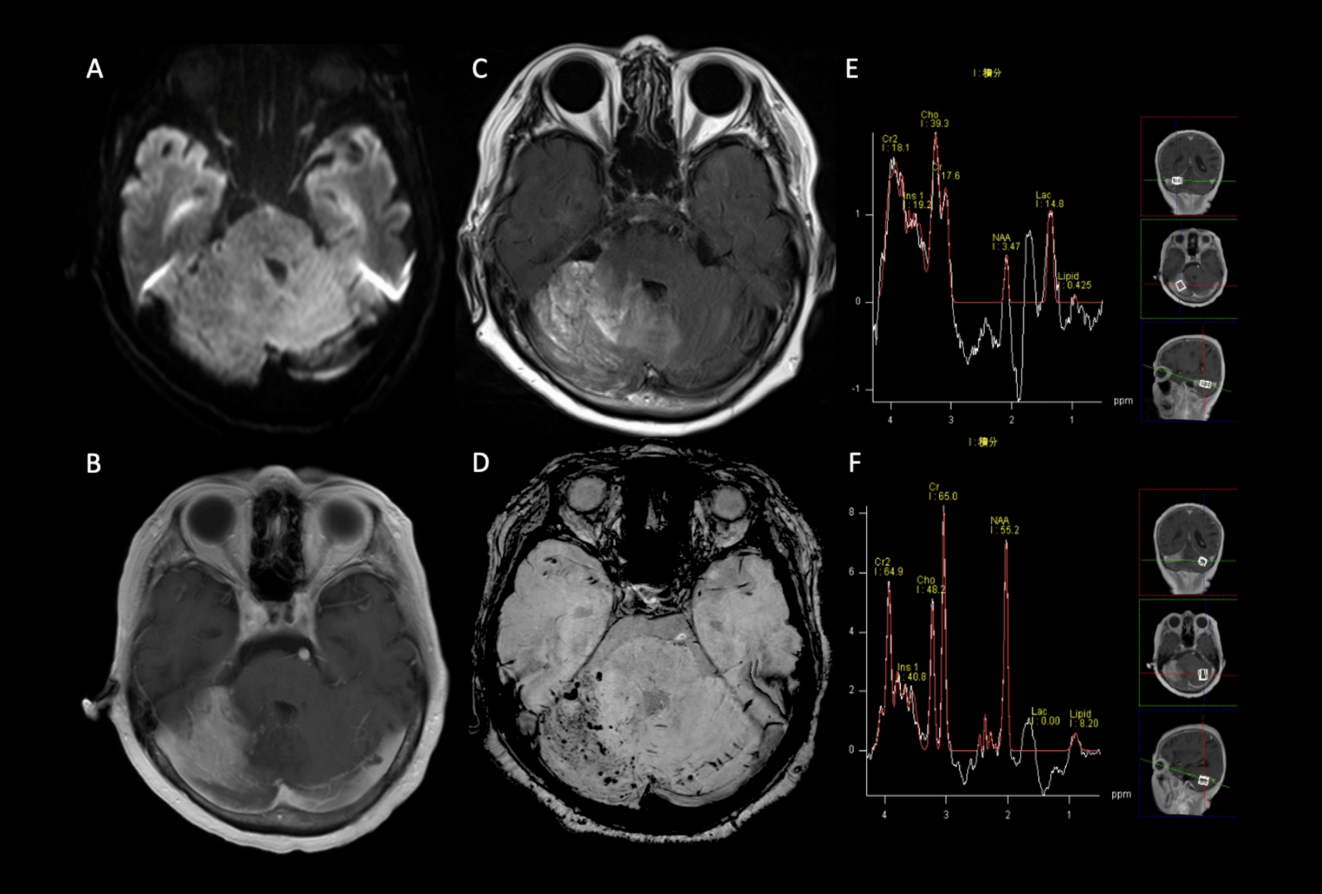
Preoperative MRI. On DWI, no infarction is observed (A), whereas on contrast-enhanced T1-weighted images and FLAIR, contrast-enhanced lesions and brain edema, respectively, are seen spreading in the right cerebellar hemisphere (B, C). SWI shows dilation of tiny veins (D). MRS indicates a relative decrease in NAA on the affected side and an increase in Lac on the affected side compared with the healthy side (E, F). MRI: magnetic resonance imaging; DWI: diffusion-weighted imaging; FLAIR: fluid-attenuated inversion recovery; SWI: susceptibility-weighted imaging; MRS: magnetic resonance spectroscopy; NAA: N-acetylaspartate; Lac: lactate

On imaging, MRI showed no high-intensity lesion on diffusion-weighted imaging (DWI), but extensive contrast-enhanced lesions in the right cerebellar hemisphere with T2/FLAIR hyperintensity around the contrast site. Susceptibility-weighted imaging (SWI) showed venous dilation centered around the contrast-enhanced area. MRS showed an increase in Lac and a decrease in NAA levels, with no left-right difference in choline-containing compounds (Figure 1).

On cerebral angiography, both tonsillar arteries were visualized from the right posterior inferior cerebellar artery (PICA), with the left tonsillar artery serving as the feeder and forming a shunt on the upper surface of the left cerebellar hemisphere. The drainage vein ran from the left superior cerebellar vein to the right superior cerebellar vein and right inferior vermian vein, and, ultimately, flowed into the lateral pontomesencephalic vein through the right petrosal vein, causing significant venous congestion in the right cerebellar hemisphere. The identification of dilation in the right petrosal vein suggested the occurrence of stenosis in the outflow pathway of the petrosal vein, leading to compromised flow in the draining vein and the progression of venous congestion. Except for the tonsillar artery, no feeders, including dural branches, were observed. The findings led to a diagnosis of venous congestion caused by a micro-AVM with a small nidus on the upper surface of the left cerebellar hemisphere, ruling out the presence of a brain tumor. Cerebral endovascular treatment was subsequently performed.

Endovascular Treatment

**Figure 2:**
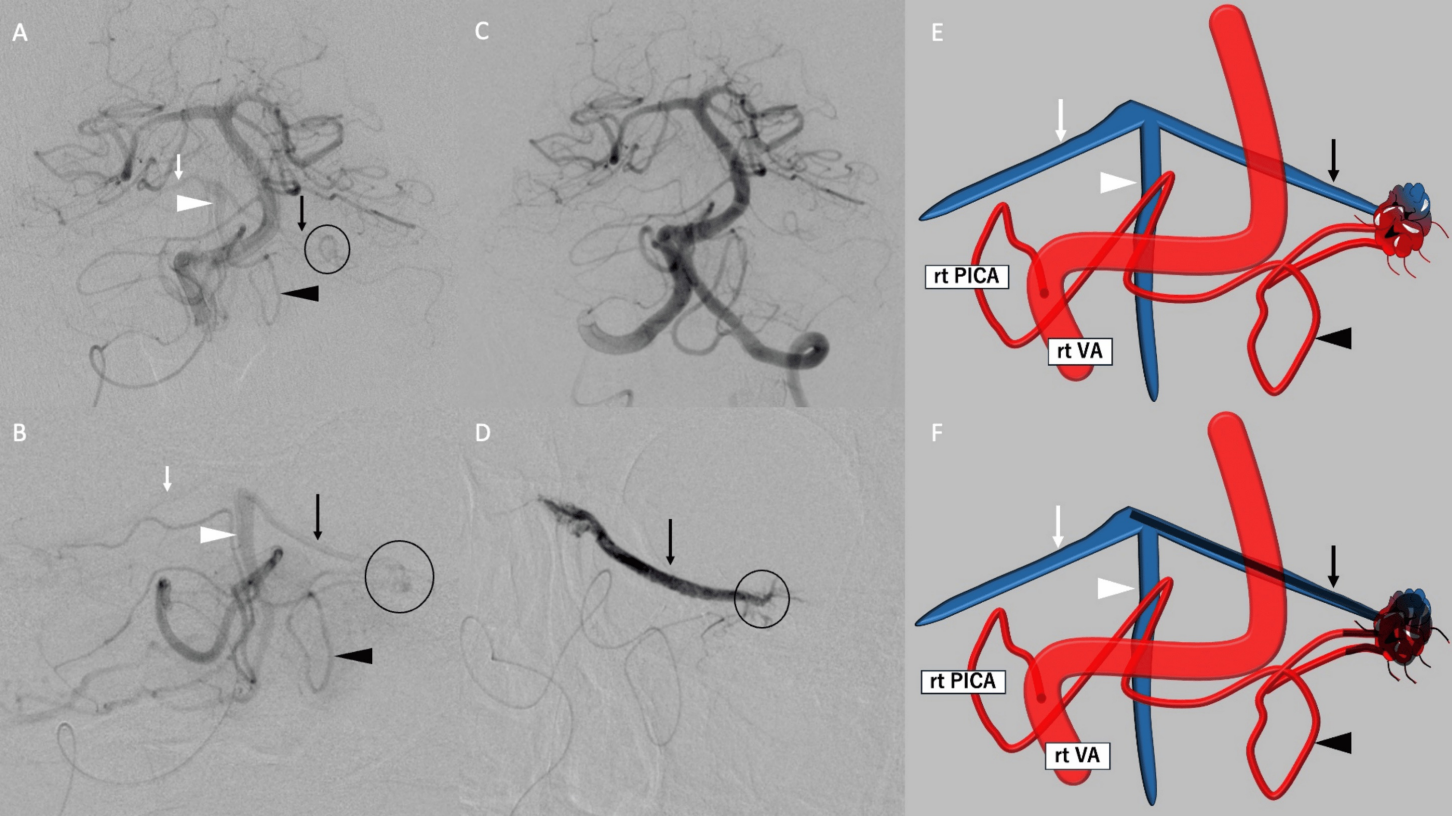
Endovascular treatment. On preoperative right vertebral artery angiography, the tonsillar arteries on both sides are seen originating from the right PICA, and a shunt is identified on the left cerebellar surface (A). Furthermore, on right PICA angiography, the shunt passes through the left tonsillar artery, draining into the right superior cerebellar vein and right inferior vermian vein through the left superior cerebellar vein (B). Post-embolization imaging shows the disappearance of the shunt (C). Early ONYX injection results in venous outflow, allowing closure of the shunt by refluxing ONYX into all feeders (D). Schema of each before and after embolization (E, F) (black arrowhead: left tonsillar artery, black arrow: left superior cerebellar vein, white arrowhead: right inferior vermian vein, white arrow: right superior cerebellar vein, circle: shunt). PICA: posterior inferior cerebellar artery

Under local anesthesia, a 5-Fr, 45-cm sheath was inserted into the right brachial artery. The 5-Fr Navien catheter, 115 cm in length, was guided to the level of the first cervical vertebra (C1) on the right vertebral artery (rt VA). The Marathon catheter was then navigated to the left tonsillar artery branching from the right PICA. Two branches originating from the terminal part of the left tonsillar artery served as feeders, and transarterial embolization (TAE) with ONYX 18 was performed. The first branch was occluded to block the feeder, and the second was intended for therapeutic embolization. Early in the injection, ONYX leaked into the venous side, but by completely backflowing ONYX into all feeders, the shunt was entirely sealed, achieving a cure for the AVM. Confirmation of no shunt visualization from other vessels marked the completion of the procedure (Figure 2).

Postoperative Course

**Figure 3:**
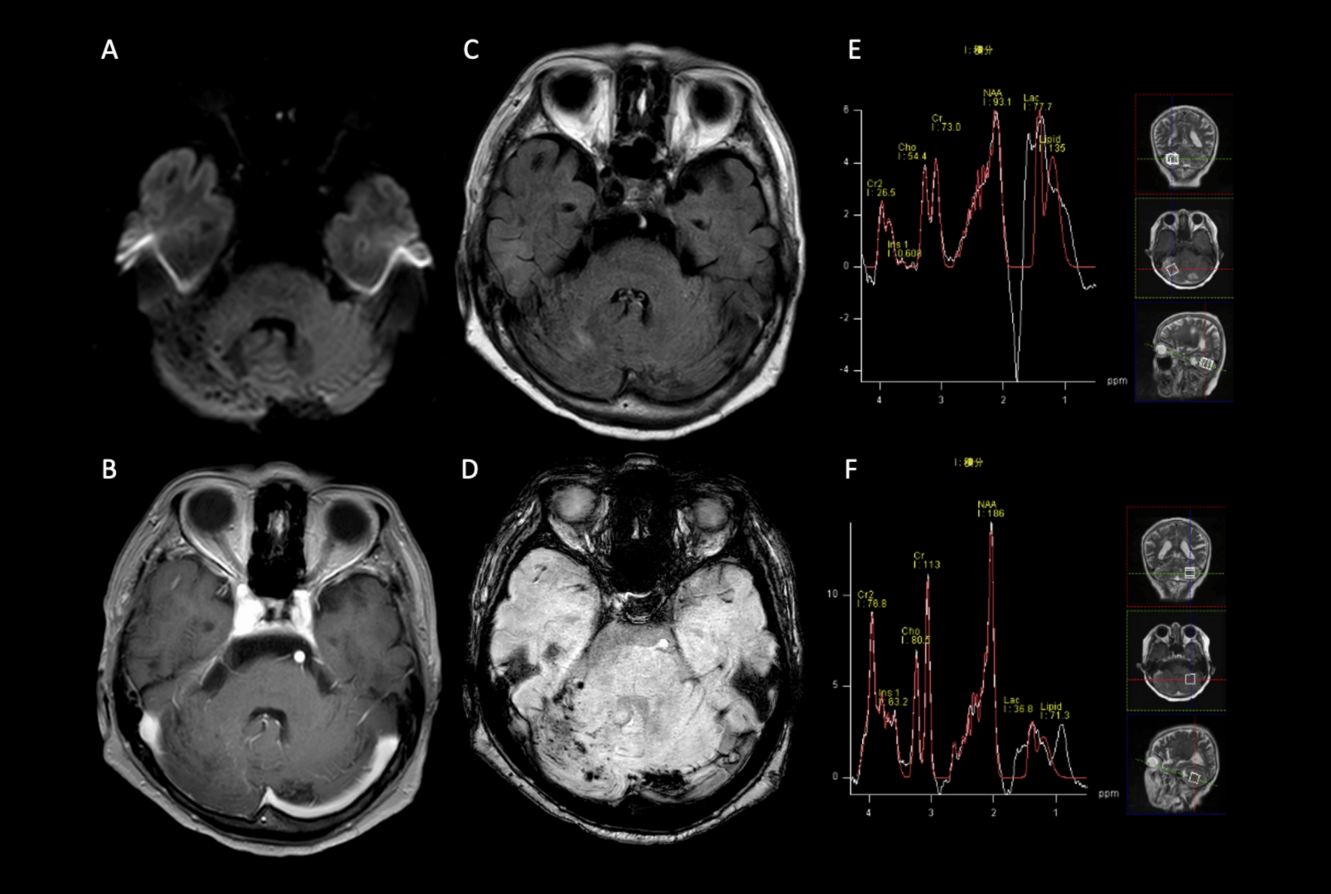
MRI 10 months after vascular treatment. Shrinkage of contrast-enhanced lesions, cerebellar hemisphere edema, and venous dilation on the right side are observed (A-D). However, there is no improvement in NAA and Lac levels which remain relatively high on the affected side (E, F). MRI: magnetic resonance imaging; NAA: N-acetylaspartate; Lac: lactate

A slight cerebral infarction was observed around the nidus in the left cerebellar hemisphere, but it was asymptomatic. Early postoperatively, improvements were noted in the contrast-enhancing and edematous lesions. Follow-up cerebral angiography performed two weeks after the surgery showed no recurrence of the shunt. On follow-up MRI, the contrast-enhanced and edematous lesions had decreased in size, and the dilated veins tended to disappear. However, MRS showed a persistent decrease in NAA and elevated Lac levels. Her dysarthria and dizziness also improved, leading to independent ambulation and discharge 30 days after admission. Follow-up in the outpatient setting showed no recurrence of symptoms. However, on MRS conducted 10 months postoperatively, a sustained decrease in NAA and elevated Lac levels were still observed (Figure 3).

Case 2

Presentation

The patient was a 59-year-old man with chief complaints of tinnitus. His medical history included diabetes and hypertension. For the past few years, the patient had been experiencing pulsatile tinnitus. MRI revealed a dural arteriovenous fistula (dAVF). He was then referred to the neurosurgery department for further evaluation.

Neurological Examination

No neurological deficits such as paralysis or aphasia were observed.

Imaging

**Figure 4:**
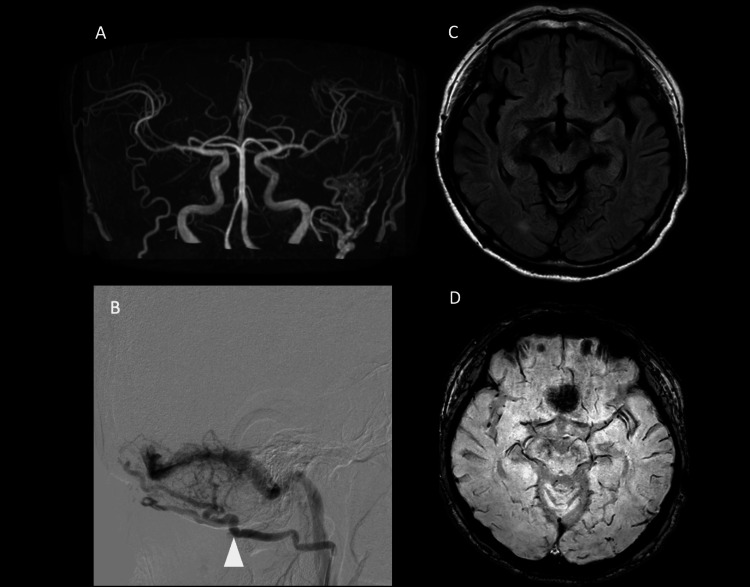
Initial MRI and cerebral angiography. MRA shows TS-SS dAVF (A). Cerebral angiography reveals a shunt formation at the transverse sinus primarily fed by the occipital artery (white arrowhead) without CVR (B: lateral view) (Cognard IIa). No FLAIR hyperintense lesions can be observed (C). SWI showed no venous dilation (D). MRI: magnetic resonance imaging; MRA: magnetic resonance angiography; TS-SS: transverse sinus-sigmoid sinus; dAVF: dural arteriovenous fistula; CVR: cortical venous reflex; FLAIR: fluid-attenuated inversion recovery; SWI: susceptibility-weighted imaging

The initial MRI did not show venous dilation on SWI, nor was there any FLAIR hyperintensity. Cerebral angiography revealed transverse sinus-sigmoid sinus dAVF, but no significant CVR was observed. This case was classified as Cognard type Ⅱa (Figure 4).

Course

**Figure 5:**
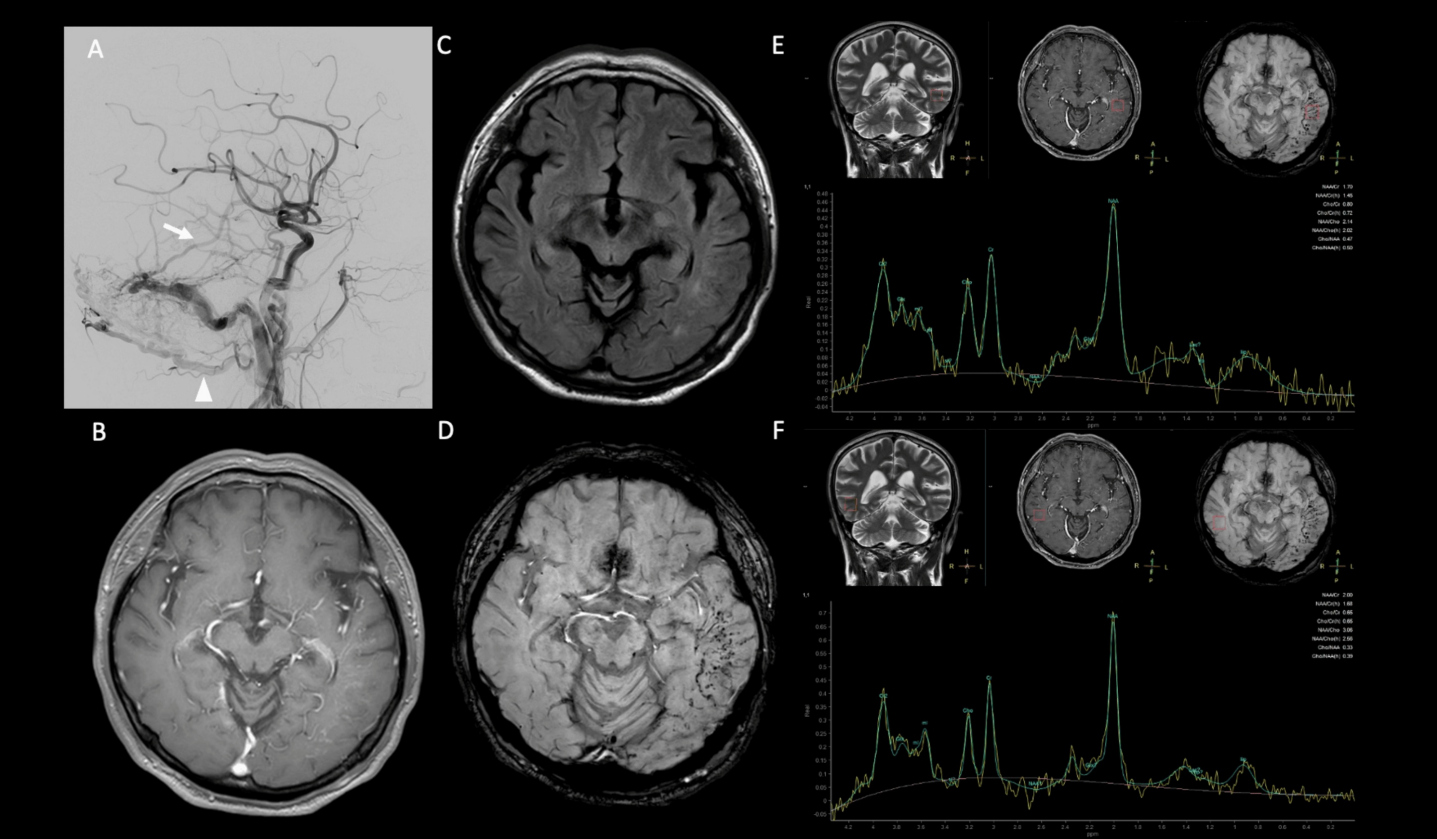
One year after observation. Cerebral angiography shows reflux into the vein of Labbe (white arrow) and the left occipital lobe cortical vein, classified as Cognard IIb (A: lateral view). Slight enhancement is observed in the left temporal lobe (B). FLAIR imaging shows a hyperintense signal in the left temporal lobe (C). SWI reveals venous dilation (D). MRS shows normal levels of both NAA and Lac (E, F) (occipital artery: white arrowhead). FLAIR: fluid-attenuated inversion recovery; SWI: susceptibility-weighted imaging; NAA: N-acetylaspartate; Lac: lactate

**Figure 6:**
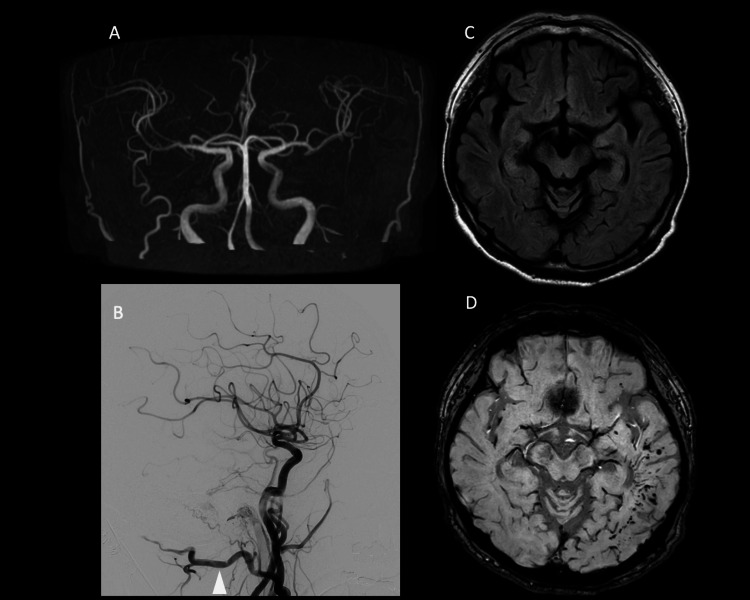
Post-treatment. MRA taken three months after treatment shows the disappearance of the dAVF (A). Cerebral angiography confirms the absence of the shunt (B: lateral view). SWI shows no significant change in venous dilation, but FLAIR imaging reveals a reduction in the hyperintense area (C, D) (occipital artery: white arrowhead). MRA: magnetic resonance angiography; dAVF: dural arteriovenous fistula; FLAIR: fluid-attenuated inversion recovery; SWI: susceptibility-weighted imaging

Due to the absence of intracranial reflux, the patient was initially placed under observation. After one year, although there was no worsening of symptoms, SWI showed progression of venous dilation in the left temporal and occipital lobes. Additionally, the MRI revealed FLAIR hyperintensity and enhanced areas in the left temporal lobe. MRS did not show an increase in NAA or Lac; however, due to the possibility of CVR, endovascular treatment was performed. Postoperatively, the tinnitus resolved, and the patient was discharged home. A follow-up MRI three months later showed a reduction in the FLAIR hyperintense area (Figures 5, 6).

## Discussion

MRS allows for the non-invasive measurement of metabolites within the body, enabling various pathological analyses [1]. MR uses the phenomenon of nuclear magnetic resonance to obtain signals, with MRI visualizing signals from hydrogen nuclei (1H: protons) present in water. The concentration of water in the brain’s white matter is approximately 35 M (=mol/L), whereas the targeted metabolites’ concentrations in MRS are around 15 mM. On MRS, water signals are suppressed through methodological adjustments, enabling data acquisition [6]. Major metabolic changes in ischemic cerebral vascular disorders include cellular respiratory impairment caused by disruption in the supply of glucose and O_2_ due to reduced blood flow. In the acute phase of cerebral infarction, anaerobic glycolysis is enhanced, leading to an early increase in Lac preceding a decrease in NAA. As ischemia progresses, oxidative phosphorylation within mitochondria halts, high-energy phosphate compounds deplete, and cell membrane potential cannot be maintained, resulting in depolarization. Consequently, impaired NAA synthesis and the release/degradation of extracellular NAA due to depolarization occur, leading to an NAA decrease. In addition, during the subacute to chronic phase, all detected metabolites decrease with tissue necrosis. MRS classifies the pathophysiology into the following three regions based on the changes in NAA and Lac: (1) ischemic core region showing irreversible changes with a significant decrease in NAA and a marked increase in Lac levels; (2) ischemic penumbra region with an increase in Lac and a mild decrease in NAA (encompassing the so-called penumbra, which may undergo selective neuronal loss over time even with relief therapy); and (3) ischemic penumbra region showing only a mild increase in Lac levels (considered reversible changes from a metabolic perspective) [3]. In a transient ischemic attack, a 10-15% reduction in NAA in the acute phase, with recovery reported 12-18 months after onset [7], and improvement in NAA after reopening following acute brachial artery occlusion have been documented [8]. Furthermore, in chronic symptomatic internal carotid artery stenosis/occlusion, reports have shown improved NAA/Cr following extracranial-intracranial bypass compared to conservative treatment [9]. Thus, MRS is deemed valuable for evaluating the degree of tissue ischemic injury and tissue reversibility during blood flow reconstruction. Regarding the persistence of Lac in the chronic phase, Houkin and colleagues proposed hypotheses, including (1) non-washout due to blood flow impairment; (2) production in the penumbra; (3) production by phagocytic cells infiltrating brain parenchyma after stroke; and (4) continuous glucose supply to damaged tissue through the development of collateral circulation [10].

In Case 1, the pronounced venous dilation observed on SWI suggests the occurrence of venous congestion due to cerebral venous reflux. The lack of improvement in NAA reduction even after shunt disappearance indicates that brain tissue may have progressed to necrosis due to venous congestion, implying an irreversible condition. Foix-Alajouanine syndrome shares a similar pathophysiology, and it is conceivable that the present case has a comparable condition [11-13]. The treatment in this case is believed to have been significant in halting the progression of the disease, given the lack of improvement in NAA levels. Although the dizziness and dysarthria caused by cerebellar hemisphere impairment became less prominent, the possibility of residual sequelae was considered high, particularly if eloquent areas were affected. However, in cases of dAVF with cortical venous reflux causing NAA reduction, there are reported cases where postoperative NAA improved [14], suggesting that reversible outcomes can occur even when NAA reduction is observed. This indicates that MRS may also be effective in evaluating treatment outcomes.

Compared with Case 2, SWI showed venous dilation, whereas T2/FLAIR high-intensity and contrast-enhanced lesions were minimal. On MRS, there was no evidence of NAA reduction or Lac elevation, suggesting that venous congestion had not yet led to necrosis. The mild changes in NAA observed in the edematous lesions of white matter in symptomatic meningioma suggest that neurological function may recover after tumor resection and that MRS may be useful in distinguishing between reversible and irreversible brain damage preoperatively [2]. Therefore, it can be expected that T2/FLAIR hyperintensity with mild NAA changes may follow a reversible course.

In cerebral venous thrombosis (CVT), venous occlusion leads to an increase in venous pressure and a decrease in capillary perfusion pressure, resulting in increased cerebral blood flow and interstitial edema. Subsequently, disruption of the blood-brain barrier leads to vasogenic edema. If collateral veins do not develop to withstand the increased venous pressure, bleeding may occur. Moreover, the elevation of venous pressure can lead to a decrease in arterial perfusion pressure, causing cytotoxic edema and ischemia [15]. CVT follows a reversible course, and cerebral venous infarcts are considered pathophysiologically distinct from arterial ischemic stroke. In brain arteriovenous shunt disease with cortical venous reflux, the annual hemorrhage rate is reported to be 1.4-1.5% in patients without a history of bleeding and 7.4-7.6% in patients with a history of bleeding [16,17]. The prognosis after bleeding is poor, making surgical intervention a consideration, but the risk of bleeding and surgical complications must be carefully weighed. However, in cases such as the present one, in which bleeding did not occur, but progression to necrosis and T2/FLAIR hyperintensity due to venous congestion occurred, additional evaluation methods such as MRS beyond assessing the extent of morphological reflux are needed. As venous congestion progresses, Lac elevation is expected due to impaired blood flow.

MRS is limited by dependence on relative evaluation without providing absolute values. Moreover, there are challenges in determining the region of interest, and capturing images is difficult due to prolonged imaging time, particularly in patients with body movement. Due to the small number of cases, it is difficult to set a cutoff value for NAA at the current time, and only relative evaluation compared to the healthy side is possible. It is crucial to examine further cases, including setting the cutoff value, to determine if MRS can predict the transition to irreversible NAA reduction in such scenarios. Despite these limitations, we believe that MRS may help understand the pathophysiology of brain arteriovenous shunt disease, including for evaluating treatment outcomes.

## Conclusions

The findings of MRS for NAA/Lac levels in AVM/Fs are believed to evaluate the degree of brain damage due to venous congestion and to be useful in predicting neurological outcomes before and after treatment. Although venous reflux in brain arteriovenous shunt disease is a risk factor for brain hemorrhage, the possibility of irreversible brain damage due to venous congestion exists even without brain hemorrhage. In cases of asymptomatic patients undergoing imaging follow-up, the addition of MRS is recommended. Further case studies are needed to establish a cutoff for the rate of NAA decrease compared to the healthy side which indicates irreversibility.
